# CircularRNA-9119 protects hepatocellular carcinoma cells from apoptosis by intercepting miR-26a/JAK1/STAT3 signaling

**DOI:** 10.1038/s41419-020-02807-0

**Published:** 2020-07-30

**Authors:** Lixue Yang, Hui Xue, Yanfu Sun, Lei Zhang, Feng Xue, Ruiliang Ge

**Affiliations:** Department of Hepatic Surgery II, Eastern Hepatobiliary Surgery Hospital, Shanghai, China

**Keywords:** Tumour-necrosis factors, Cancer immunotherapy

## Abstract

Hepatocellular carcinoma (HCC) is a more common malignancy than the majority of cancers and ranks second in the world’s top causes of cancer-related mortality. The objective of the study was to investigate and explain how circularRNA-9119 (circ9119) regulated the properties of HCC cell lines. Cancer cells isolated from HCC patients and HCC cell lines showed clearly upregulated expression of circ9119 and Janus kinase 1 (JAK1) with decreased levels of miR-26a compared to healthy controls and normal hepatic cells. To determine the function of circ9119, circ9119 was silenced in HCC cells, resulting in significantly less proliferation of HCC cells and increasing apoptosis. Circ9119 silencing also resulted in the upregulation of miR-26a. Bioinformatics prediction and dual-luciferase reporter assays showed that circ9119 targeted miR-26a. Further studies revealed that miR-26a had the opposite effect on circ9119; the inhibition of miR-26a antagonized circ9119 silencing, leading to reduced cell proliferation and increased apoptosis, while the ectopic overexpression of miR-26a impaired cell growth. Additionally, we found that the JAK1 3′-UTR was targeted by miR-26a; a decrease in the levels of JAK1 protein and mRNA followed transfection of a miR-26a mimic. Administration of the JAK1 inhibitor, baricitinib, caused the activation of signal transducer and activator of transcription 3 (STAT3) and revealed an effect similar to that of circ9119 silencing on cell proliferation and apoptosis. These results showed that circ9119 could modulate apoptosis, and broadly, cell proliferation by competitively binding miR-26a, which targeted JAK1-STAT3, in HCC cell lines. This study is a novel description of circ9119 regulation of HCC.

## Introduction

Among populations diagnosed and living with cancer, those with primary liver cancer form one of the largest populations and contribute to most cancer deaths. Histologically, hepatocellular carcinoma (HCC) is a highly invasive subtype of primary liver cancer with metastatic potential and a low survival rate. It forms 75–85% of all primary liver cancers^1^. HCC is rarely confirmed in the early stage, when its symptoms and biomarkers are almost latent or too insignificant to be identified, and is instead diagnosed in advanced stages, lowering the cure rate. More so, those who have undergone hepatectomy or liver transplantation are frequently subject to survival declines due to recurrence and metastasis^2^. Diagnosis and prediction of the course of HCC using new biomarkers and treatment options is urgently needed for improved survival and quality of life.

Circular RNAs (circRNAs) are a type of non-coding RNA (ncRNA) that feature microRNA (miRNA) response elements that can bind to messenger RNAs (mRNAs), facilitating recent evidence of their potential to regulate genes^3^. As molecular sponges of miRNAs, circRNAs derepress miRNA target genes, affecting post-transcriptional regulation^4^. Primary factors contributing to the low survival among HCC patients include the absence of working biomarkers and the presence of classical biomarkers, such as des-gamma-carboxyprothrombin (DCP), alpha-fetoprotein-L3 (AFP-L3), and alpha-fetoprotein (AFP), which are poorly effective in accurately diagnosing early HCC^5^. A recent report demonstrated that miRNAs and long non-coding RNAs (lncRNAs) play roles in the diagnosis of HCC as potential biomarkers^6,7^. The potential of circRNAs for the biological diagnosis and prognosis of HCC has been identified. Previous findings also reveal circRNA regulation of biological processes, varying from the epithelial-mesenchymal transition to invasion and metastasis, to cell proliferation and cell death, through which circRNAs exert oncogenicity or suppression of HCC progression^2^. Dysregulation of the expression of some circRNAs precipitates HCC development via its role in proliferation, invasion, migration, programmed cell death, and other biological processes in HCC cells. For instance, besides being involved in HCC cell proliferation, circCDYL contributes to chemoresistance and stem cell-like properties, circDYNC1H1 regulates the migration of HCC cells, and circ0016788 is involved in the established process in which the cells invade the body and commit suicide, whereas circZNF652 accelerates the migration and invasion of HCC cells^8–11^. miRNAs exert their regulatory function on gene expression through the binding of the 3′-UTR of miRNA-targeted mRNAs^12^. A couple of circRNAs have been reported to sponge miRNAs and function in disease development by regulating miRNA transcription^13^. The contribution of miRNA to HCC progression has also been shown, specifically for miR-122, a major player in hepatic function and metabolic equilibrium, ranking first in liver content and making up 70% of the total liver miRNA pool^14,15^. The involvement of miR-221/222 and miR-214 in carcinogenesis, especially in HCC progression, has been established. MiR-221/222 modulates cell proliferation and cell cycles across different tissues, whereas miR-214 modulates the phosphatase and tensin homolog (PTEN)/Akt, tyrosine kinase receptor and β-catenin signaling pathways^16,17^.

CircularRNA-9119 (circ9119) is specifically expressed in the receptive endometrium at a high expression level (NCBI/Gene Bank accession number GSE85384). Moreover, circ9119 shared miRNA response elements with miR-26a, which directly downregulates PTGS2^18^. Our aim was to confirm the position(s) of circ9119 and miR-26a during HCC progression by comparing their expression profiles between HCC and non-HCC specimens and cells. Further exploration showed circRNA-miRNA links to essential signaling pathways, such as Janus kinase 1 (JAK1) and signal transducer and activator of transcription 3 (STAT3).

## Material and methods

### Human tissue specimens

With eligibility verified by specialized pathologists, 20 HCC tissue specimens and adjacent normal tissue were collected at the Eastern Hepatobiliary Surgery Hospital. All samples were frozen in liquid nitrogen before qPCR analysis and western blotting. Patients who had undergone actinotherapy, radio frequency ablation and chemotherapy were excluded from the surgery. Cancer staging was in line with the American Joint Committee on Cancer 7th edition staging system (2010). The trials were approved by the Ethics Committee of the Eastern Hepatobiliary Surgery Hospital and informed consent was obtained from all subjects.

### Cell lines and cell culture

Human HCC cell lines, including HepG2, SMMC-7721, BEL-7402, and Huh-7, were purchased from the Cell Bank of Type Culture Collection of the Chinese Academy of Science (CAS), and HEK293T cells, from the American Type Culture Collection (ATCC). The L02 immortalized normal healthy hepatic cell (HHC) line was purchased from the Institute of Biochemistry and Cell Biology, CAS, China. In a 5% CO_2_ moisturized incubator, cells were maintained at 37 °C in high glucose Dulbecco’s modified Eagle’s medium (DMEM) with 1% non-essential amino acids, 1 mM sodium pyruvate, 1 mM l-glutamine, and 10% fetal bovine serum (FBS).

### Vector construction

miRNA binding site sequences in JAK1 and circRNA-9119 were analyzed using miRanda version 3.3a (https://bioweb.pasteur.fr/packages/pack@miRanda@3.3a, verified 22 June 2018) and Targetscan ver. 6.2 (http://www.targetscan.org/, verified 22 June 2018), to distinguish genes that were targeted by miR-26a. Luciferase assays were preceded by the cloning of the JAK1 3′-UTR and the entire circRNA-9119, in which the miR-26a target site was identified, and insertion into the psiCHECK-2 vector (Promega) to construct mutated plasmids. The full length circRNA-9119 (synthesized by Genscript, Beijing) was inserted into the pCD2.1-ciR vector (Geneseed Biotech, China) prior to reporter construction for assaying gene overexpression.

### Cell transfections

The siRNA-circ9119 (si-circ9119), siRNA-NC (si-NC), miR-26a inhibitor, NC inhibitor, miR-26a mimic, and NC mimic (GenePharma, Shanghai, China) were transfected into HepG2 and Huh-7 cells. Their sequences are displayed as follows: si-circ9119, 5′-UAU CCA AUG CUA GCA GUU CAG G-3′; si-NC, 5′-CCG CTC GAG CTA GTG GGA CGC GGA CAT-3′; miR-26a mimic, 5′-UUC AAG UAA UCC AGG AUA GGC U-3′; NC mimic, 5′-UUG UAC UAC ACA AAA GUA CUG-3′; miR-26a inhibitor, 5′-UUC AAG UAA UCC AGG AUA GGC U-3′; and NC inhibitor, 5′-UUG UAC UAC ACA AAA GUA CUG-3′. Transfections were conducted using the Lipofectamine 2000 reagent (Invitrogen, Carlsbad, CA, USA).

### Dual luciferase reporter assay

To culture HEK293T cells, the circ9119/JAK1 wild-type (psiCHECK2-WT) or miR-26a-mimic transfected mutant (psiCHECK2-MU) plasmids were first cotransfected into HEK293T cells. The DualGlo luciferase assay system (Promega) was initiated at 36 h post-transfection to assay Firefly and Renilla luciferase activity^19^. Three trial cycles were required.

### Real-time quantitative polymerase chain reaction (qRT-PCR)

Total RNA isolation using the TRIzol reagent was performed. After removing DNA contamination from the RNA solution by using RNase-free DNase I (Invitrogen), the obtained product was verified via *GAPDH* PCR amplification, followed by reverse transcription of total RNA (2 µg). qRT-PCR was performed with the Applied Biosystems Power SYBR^®^ Green PCR Master Mix kit and the Applied Biosystems 7300 Real-Time PCR System (Foster City, US). The sequences of primers are as follows: circ9119 Forward: 5′-CCG TGG GTT TGC TGA CCA TT-3′, circ9119 Reverse: 5′-GAC TCC ACG AAA TCG GCC TC-3′; miR-26a Forward: 5′-GCG CTT CAA GTA ATC CAG-3′, miR-26a Reverse: 5′-GTG CAG GGT CCG AGG T-3′; *JAK1* Forward: 5′-CCC CCA TTG ATC GTC CAC AA-3′, *JAK1* Reverse: 5′-CAC ATA CAT CCC CTC CTC GC-3′; *GAPDH* Forward: 5′-TAG TGA AGC AGG CAT CGG AG-3′, *GAPDH* Reverse: 5′-CGA AGG TGG AAG AGT GGG TG-3′. This was followed by comparative 2^−ΔΔCT^ analysis as specified in the Applied Biosystems User Bulletin No. 2-P/N 4303859 to quantify expression relative to transcripts^20^.

### Western blotting (WB)

The protein bands were electrically transferred onto polyvinylidene difluoride membranes following protein (15 μg per well) separation on a 12% gel via SDS-PAGE. Membranes were blocked for 1 h with a 5% solution of nonfat powdered milk in tris-buffered saline (TBS) at room temperature, and incubated with primary antibodies at 4 °C. The membranes were incubated with secondary antibodies and rinsed twice using TBS with 0.1% Tween-20 before observation of the antigen-antibody complex via the ECL detection kit (Zhongshan Biotechnology). β-Actin was used as a control.

### MTT assay

An 3-(4,5-dimethylthiazol-2-yl)-2,5-diphenyltetrazolium bromide (MTT) assay was performed to evaluate cell survival. Briefly, cells were treated with 20 μL MTT (0.5 mg/mL), and the supernatant was discarded. Dimethyl sulfoxide (DMSO, 150 μL) was then added to each well, with rotation for 10 min, to dissolve the formazan dye. An Infinite M200 microplate reader (Tecan, Männedorf, Switzerland) was used to measure the absorbance at 540 nm.

### Colony generation assay

Cells were transfected using various reagents. Cells were resuspended in DMEM supplemented with 10% FBS after two days of transfection and plated on an 8-mm layer of 0.4% top agar, followed by transfer into 12-well plates containing 0.5 mL of 0.5% bottom agar. After 14 days, four regions were randomly chosen from each plate and colonies were quantified.

### Immunofluorescence assay and confocal microscopy

An immunofluorescence assay was performed upon 16 h cell culture at 50% confluence. Cells were fixed and permeabilized at room temperature in 100% methanol for 15 min. The slides were rinsed multiple times with PBS for rehydration. Bovine serum albumin (1%) in PBS was used to block nonspecific binding sites. The cells were rinsed thrice in PBS and probed with fluorescent (DyLight 594 or fluorescein isothiocyanate (FITC))-conjugated secondary antibodies (1:100 dilution, incubated at 4 °C for 16 h). Cell nuclei were counterstained with 4′,6-diamidino-2-phenylindole (DAPI) and the samples were analyzed by a confocal laser scanning microscope (Leica TCS SP5, Wetzlar, Germany).

### Apoptosis detection

Cell apoptosis was evaluated using flow cytometry (FCM) with an Annexin V-FITC/propidium iodide (PI) apoptosis detection kit (BD Pharmingen™). A cell suspension was prepared in 20 µL binding buffer, followed by treatment in 10 µL Annexin V-FITC and 5 µL PI. The apoptotic rate of cells was measured using FCM.

### JAK1 inhibitor treatment

Baricitinib, a JAK1 inhibitor (S2851, Selleckchem), was utilized to block JAK1-STAT3 signal transduction. Cells were incubated with 2 μM baricitinib for 1 h.

### Animal tests

BALB/c-nu mice (female, aged five weeks) were purchased from Vital River (Beijing, China). Huh-7 cells (1 × 10^6^) were infected with 1 × 10^7^ transduction units (TU) lentiviral particles carrying si-NC or si-circ9119. All mice were subcutaneously injected with these cells through the right oxter following acclimatization for three days. The weights of tumors were expressed in grams, and the formula (L × W^2^)/^2^ was used to calculate the volume of tumors. At day 29 after injection, mice were sacrificed. All animal tests were carried out under the approval of the Institutional Animal Care and Use Committee of the Eastern Hepatobiliary Surgery Hospital.

### Statistical analysis

Statistical analysis was completed using SPSS Statistics 17.0. *t*-tests or one-way ANOVA were conducted to estimate differences, and further analyses were carried out by the least significant difference method. A statistically significant difference was considered at a two-tailed *P* < 0.05, and a highly significant difference at *p* < 0.01.

## Results

### Circ9119 was expressed in HCC specimens and cell lines

**Fig. 1:**
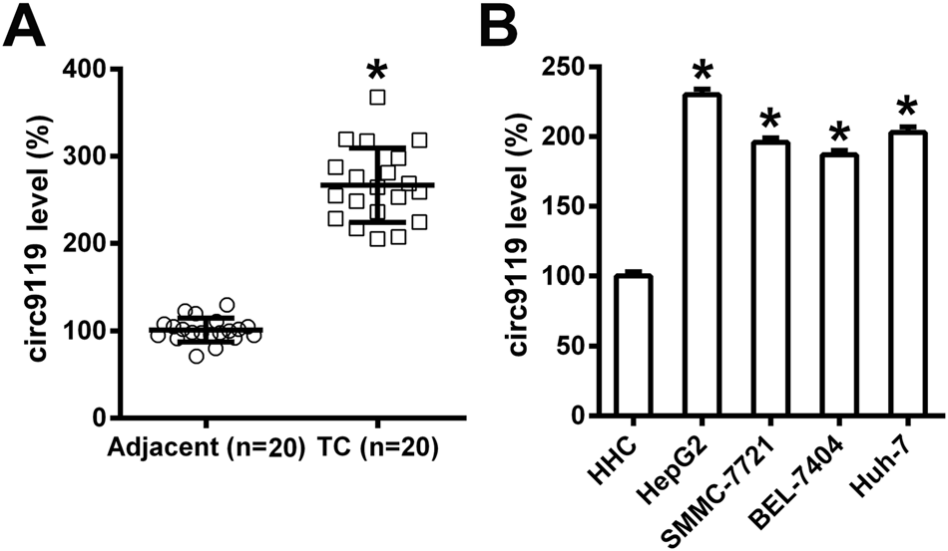
Circ9119 is upregulated in HCC tissue specimens and cell lines. **a** qRT-PCR shows circ9119 expression levels in HCC specimens (*N* = 20) and in specimens from adjacent healthy tissue (*N* = 20). **b** qRT-PCR shows circ9119 expression in L02 immortalized normal healthy hepatic cells (HHC) and four HCC cell lines, namely Huh-7, SMMC-7721, HepG2 and BEL-7404. *p < 0.05 vs. indicated groups.

circ9119 expression in 20 HCC samples and 20 adjacent normal tissues (Table 1) as analyzed via qRT-PCR. Circ9119 was more highly expressed in HCC samples than in controls (Fig. 1a). In addition, circ9119 was upregulated in human HepG2, BEL-7404, SMMC-7721, and Huh-7 (HCC cell lines), compared with normal healthy hepatic cells (HHCs) (Fig. 1b), suggesting that circ9119 is involved in HCC development.

**Table 1:** Relationship between circ9119 expression and clinical-pathologic features of HCC Patients (*N* = 20).

Features	Relative circ9119 expression levels		
	circ9119 low (*N* = 8)	circ9119 high (*N* = 12)	*p* value
*Age*			
≤50	4	5	0.682
>50	4	7	
*Gender*			
Male	6	9	0.560
Female	2	3	
*AFP (μg/L)*			
≤20	3	7	0.490
>20	5	5	
*HBV*			
Positive	6	7	0.508
Negative	2	5	
*Liver cirrhosis*			
Yes	7	11	0.756
No	1	1	
*Tumor diameter (cm)*			
≤5	6	1	0.023
>5	2	11	

HCC patients were divided into circ9119 “High” group (relative fold change was higher than the median) and “Low” group (relative fold change was lower than the median).

Differences among variables were assessed by *χ*^2^ or Fisher’s exact *χ*^2^ test.

*HBV* Positive hepatitis B virus surface antigen positive, *AFP* alpha-fetoprotein.

### Circ9119 silencing inhibited rapid proliferation of HepG2 and Huh-7 cells, but induced their apoptosis

**Fig. 2:**
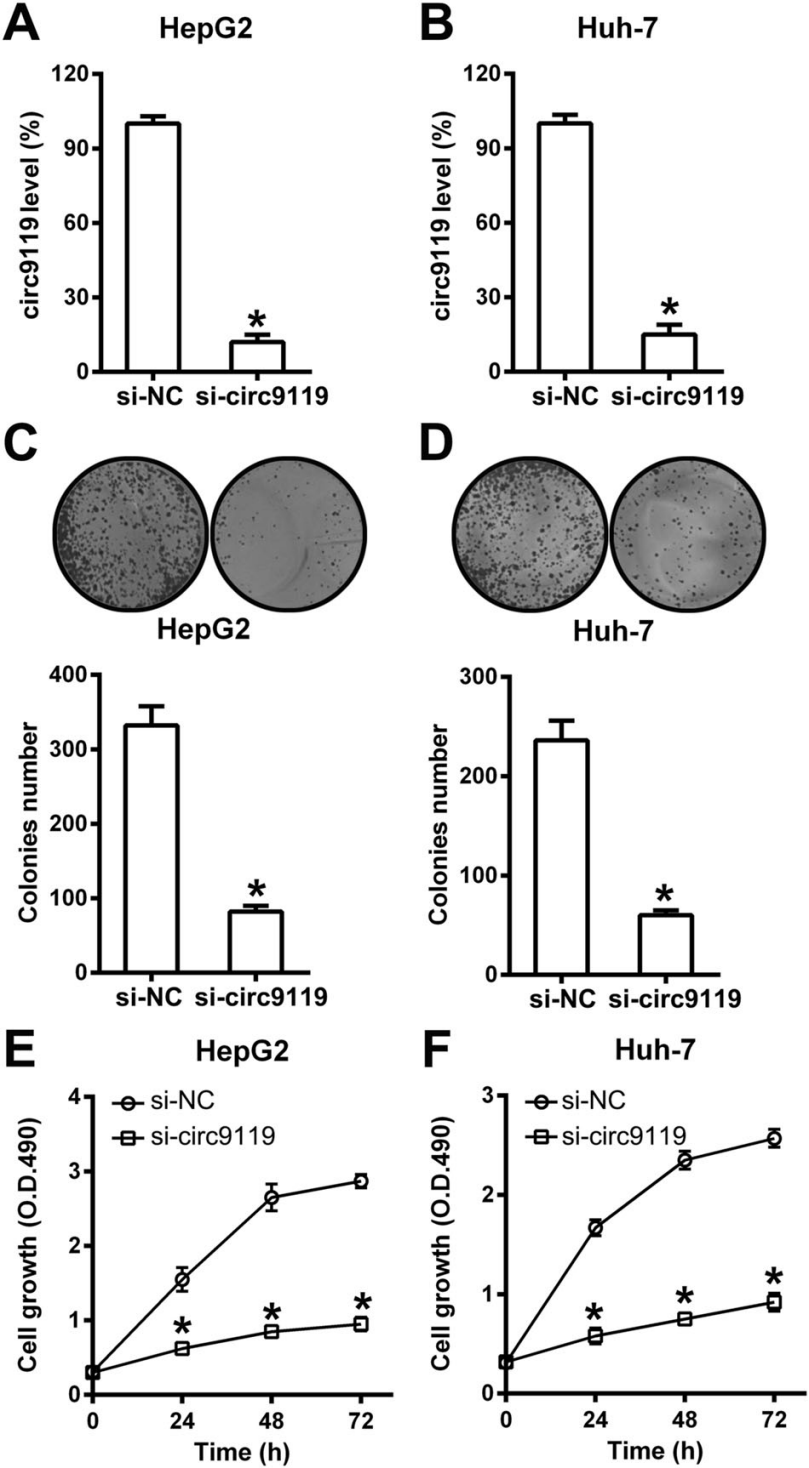
Circ9119 silencing reduces HepG2 and Huh-7 cell viabilities. Both cell lines were transfected with si-circ9119 or si-NC for 24 h. **a**, **b** qRT-PCR analysis demonstrates circ9119 levels in HepG2 and Huh-7 cell lines. **c**, **d** Soft agar colony formation assay for HepG2 and Huh-7 cells with circ9119 silencing at 72 h post transfection. **e**, **f** MTT assay reveals that transfection with si-circ9119 inhibits HepG2 and Huh-7 cell proliferation at 24, 48, and 72 h post transfection. *p < 0.05 vs. siNC group.

The function of circ9119 in the viability and proliferation of HepG2 and Huh-7 was then investigated. A significant reduction in circ9119 levels was observed after cells were transfected with circ9119 siRNA (Fig. 2a, b). Colony formation assays showed that circ9119 silencing significantly reduced the total number of colonies formed by HepG2/Huh-7 (Fig. 2c, d). MTT cell proliferation assay showed greatly lowered HepG2 and Huh-7 cell proliferation rates at 24, 48, and 72 h after transfection of the circ9119 siRNA compared to cells transfected with the siNC control (Fig. 2e, f).

**Fig. 3:**
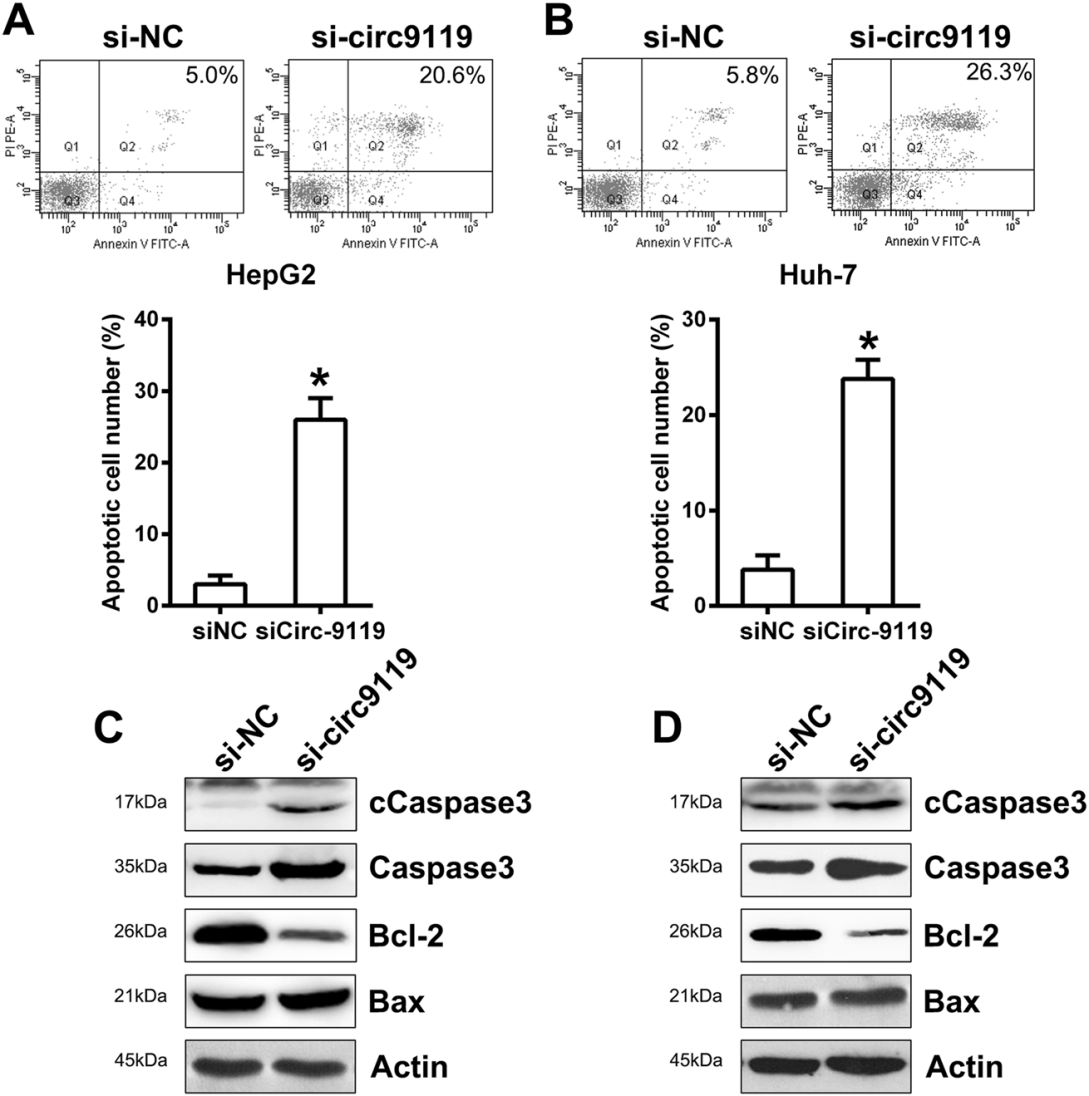
Circ9119 silencing induces HepG2 and Huh-7 cell apoptosis. Both cell lines were transfected with si-circ9119 or si-NC for 36 h. **a**, **b** FCM estimates of the total apoptotic cells in HepG2 and Huh-7 cell lines. The number represents the sum of early and late apoptotic cell percentages. **c**, **d** WB analyses indicate Bcl-2/Bax and Caspase-3/cleaved Caspase-3 expression in HepG2 and Huh-7 cells. *p < 0.05 vs. siNC group.

Because circ9119 silencing suppressed survivability and proliferation of both HepG2 and Huh-7 cells, circ9119 was hypothesized to regulate HCC cell apoptosis. In contrast with the siNC groups, HepG2 and Huh-7 cells that received circ9119 siRNA transfection showed significant increase in apoptotic cells following Annexin V-FITC & PI FCM (Fig. 3a, b). Investigation of the role of circ9119 in caspase-3 cleavage, Bcl-2, and Bax expression showed that pro-apoptotic Bax was expressed roughly at the same level under different transfection conditions, but anti-apoptotic Bcl-2 decreased with circ9119 silencing, compared to that in the siNC group. As classical biomarkers of cell apoptosis, total and cleaved caspase-3 levels were significantly highly expressed in the circ9119 silencing group (Fig. 3c, d), suggesting that circ9119 silencing-induced HepG2 and Huh-7 cell apoptosis.

### Circ9119 targeted miR-26a

**Fig. 4:**
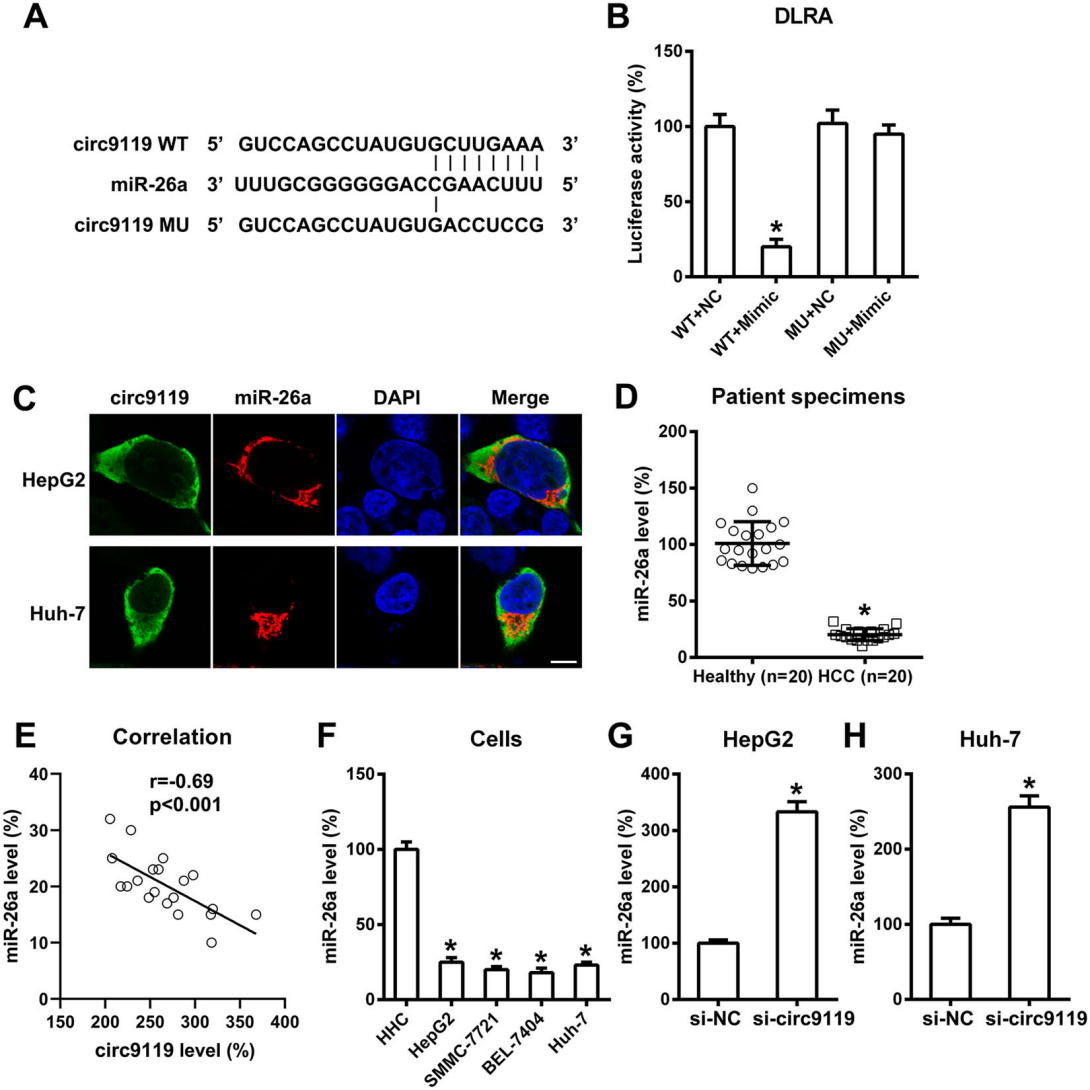
Circ9119 targets miR-26a. **a** Bioinformatics analysis showing an miR-26a binding site in the circ9119 RNA sequence. **b** Dual luciferase reporter assay results after co-transfection of a luciferase reporter containing either wild-type (WT) or mutated (MU) circ9119, and an miR-26a mimic into HEK293T cells. **c** Fluorescence of circ9119 and miR-26a in HepG2 and Huh-7 cells. Green, circ9119; Red, miR-26a; Blue, DAPI. Scale bars: 10 μm. **d** qRT-PCR results showing miR-26a levels in HCC specimens (*N* = 20) and paired contiguous healthy controls (*N* = 20). **e** Correlation analysis between circ9119 and miR-26a expression in tumor tissues from 20 HCC cases (*p* < 0.001). **f** qRT-PCR analysis showing miR-26a expression in HHC and HCC cell lines, including SMMC-7721, HepG2, BEL-7404, and Huh-7. **g**, **h** qRT-PCR results showing miR-26a levels in all groups after Si-circ9119 or si-NC transfection in HepG2 and Huh-7 cell lines, respectively. *p < 0.05 vs. indicated groups.

In previous studies, miR-26a has been shown to inhibit tumor cells in HCC^21–23^ and to be targeted by circ9119^24^. Here, bioinformatics prediction tools indicated that circ9119 gene included putative binding sites complementary to the miR-26a seed region (Fig. 4a). A dual luciferase reporter assay was conducted to investigate the link between miR-26a and circ9119 (Fig. 4b). Luciferase activity was suppressed by 76% in HEK293T cells that underwent transfection with the miR-26a mimic and that were bound to WT circ9119.

We therefore hypothesized that circ9119 also acted as an endogenous miR-26a sponge in HCC specimens and cell lines. First, confocal data indicated co-localization of circ9119 and miR-26a in both HepG2 and Huh-7 cells (Fig. 4c). Then, miR-26a expression in HCC specimens and adjacent healthy controls was examined. qRT-PCR data indicated downregulated miR-26a expression in tumor specimens compared to the controls (Fig. 4d). Further statistical analysis showed that the expression levels of circ9119 and miR-26a in HCC tumor tissues were positively related (*p* < 0.001; Fig. 4e). Additionally, miR-26a was less expressed in HepG2, SMMC-7721, BEL7404, and Huh-7 cells compared with that in HHCs (Fig. 4f). In addition, HepG2 and Huh-7 cells undergoing si-circ9119 transfection showed significantly elevated levels of miR-26a, compared with the siNC group (Fig. 4g, h). These data suggested that miR-26a was targeted by circ9119 in HCC cells.

### Role of miR-26a on circ9119-mediated HepG2/Huh-7 proliferation

**Fig. 5:**
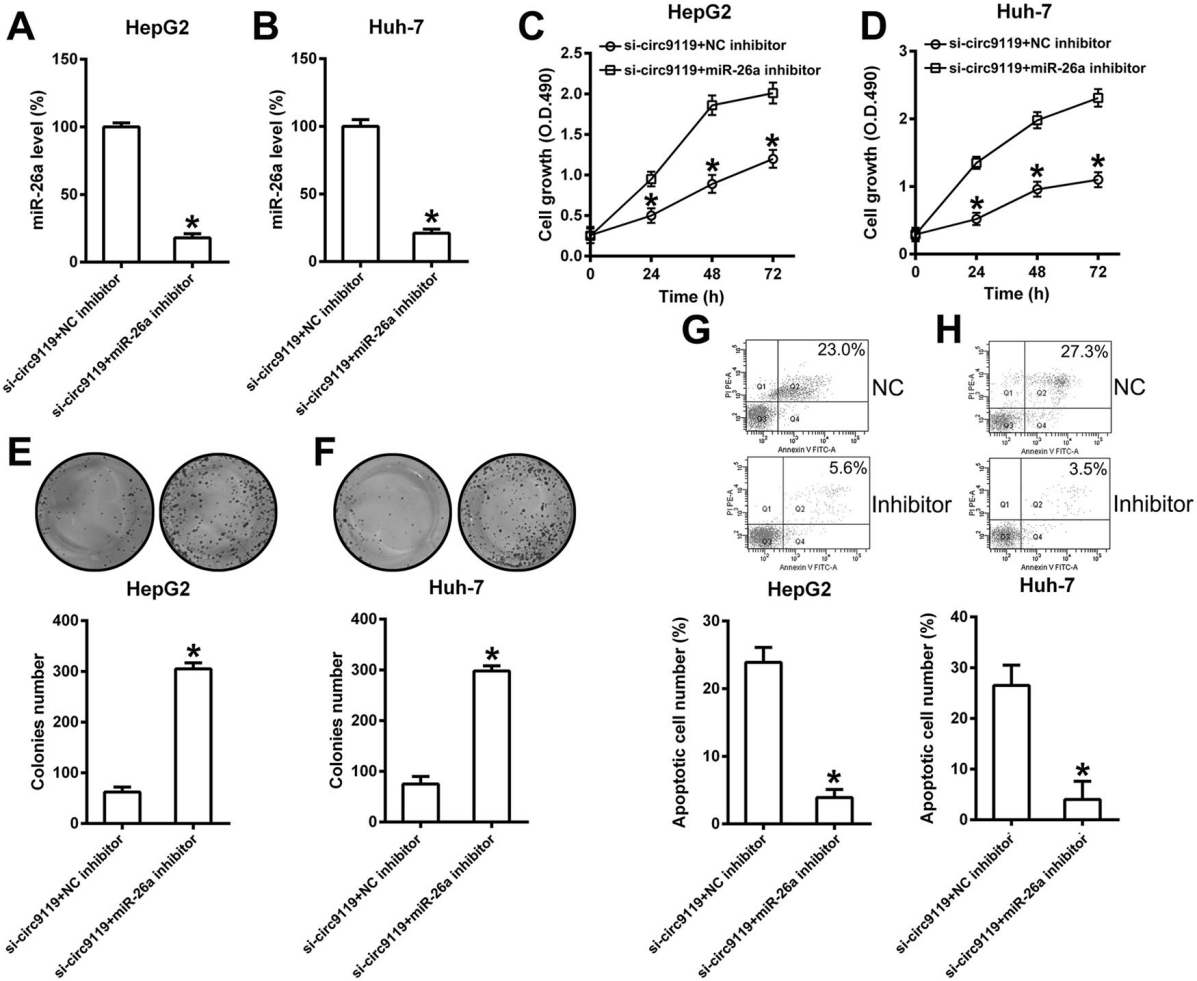
MiR-26a is an inhibitor that counteracts the effect of circ9119 silencing on the viability and apoptosis of HepG2/Huh-7 cells. Cells simultaneously underwent transfection with si-circ9119 and an miR-26a inhibitor (or NC inhibitor) for 36 h. **a**, **b** qRT-PCR analysis showing miR-26a expression in each group. **c**, **d** MTT assay showing HepG2 and Huh-7 proliferation rates, under different transfection conditions. **e**, **f** Colony formation assay indicating HepG2 and Huh-7 cell growth rates, under various transfection conditions. **g**, **h** FCM analysis showing the number of apoptotic cells following different treatments of HepG2 and Huh-7 cell lines. *p < 0.05 vs. indicated groups.

To elucidate the role of miR-26a in HepG2/Huh-7 cell proliferation, these cells were first simultaneously transfected with si-circ9119 and an miR-26a inhibitor or NC inhibitor. qRT-PCR data confirmed significantly reduced levels of miR-26a in the miR-26a inhibited groups, compared with the NC inhibited groups (Fig. 5a, b). Furthermore, the role of miR-26a inhibition in cell proliferation and apoptosis was evaluated. As shown by the MTT assay, circ9119 silencing significantly improved HepG2/Huh-7 cell proliferation during miR-26a inhibition, compared with the NC inhibitor group (Fig. 5c, d). Colony formation assay demonstrated that miR-26a inhibition restored proliferation of HepG2/Huh-7 cells inhibited by circ9119 silencing (Fig. 5e, f). Additionally, FCM results showed that transfection with the miR-26a inhibitor caused an obvious reduction in HepG2/Huh-7 apoptosis, counteracting the circ9119 silencing-induced apoptosis (Fig. 5g, h). Thus, these data suggested that circ9119 influenced the viability and apoptosis of HepG2/Huh-7 cells by regulating miR-26a levels.

### MiR-26a function was associated with the JAK1-STAT3 signal axis

**Fig. 6:**
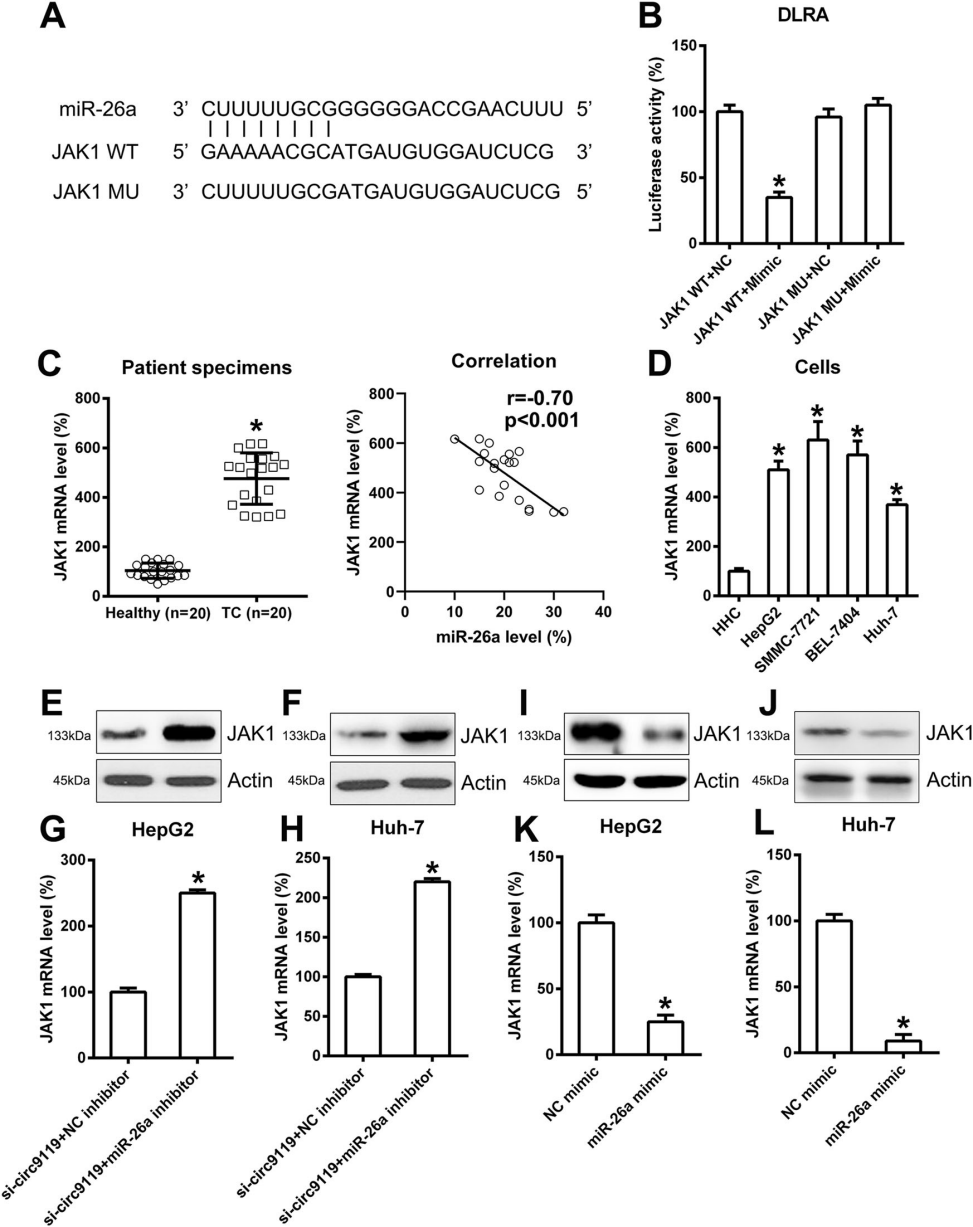
miR-26a targets the 3′-UTR of the JAK1 mRNA. **a** Bioinformatics analysis showing an miR-26a binding site in the JAK1 3′-UTR. **b** Dual luciferase reporter assay results following co-transfection of a luciferase reporter containing either wild-type (WT) or mutated (MU) JAK1 mRNA, and an miR-26a mimic into HEK293T cells. **c** qRT-PCR analysis showing JAK1 mRNA expression in HCC patients (*N* = 20) and healthy controls (*N* = 20). Correlation analysis between miR-26a and JAK1 mRNA expression in tumor tissues from 20 HCC cases (*p* < 0.001). **d** qRT-PCR showing JAK1 expression levels in HHCs and HCC cell lines (—). **e**–**h** HepG2 and Huh-7 cell lines transfected with both si-circ9119 and an miR-26a inhibitor (NC inhibitor) for 36 h. qRT-PCR and western blotting (WB) results showing JAK1 expression in each group. **i**–**l** HepG2 and Huh-7 cell lines after transfection with an miR-26a mimic or NC mimic. qRT-PCR and WB analyses showing JAK1 levels in each group. *p < 0.05 vs. indicated groups.

We then performed bioinformatics prediction to determine the putative target of miR-26a. The predictive analysis indicated miR-26a binding sites on the 3′-UTR of JAK1 (Fig. 6a). Dual luciferase reporter assay results further showed that JAK1 was essentially targeted by miR-26a, and transfection of an miR-26a mimic could also inhibit the downregulation of luciferase activity via WT JAK1 (Fig. 6b). We then determined the JAK1 expression level in HCC and healthy specimens. qRT-PCR provided evidence of the obvious upregulation of JAK1 mRNA expression in HCC specimens instead of controls (Fig. 6c). Further statistical analysis showed that the expression levels of miR-26a and JAK1 mRNA in HCC tumor tissues were positively correlated (*P* < 0.001; Fig. 6c). qRT-PCR was also carried out to compare JAK1 expression in HHC cells and four HCC cell lines, namely SMMC-7721, HepG2, Huh-7, and BEL-7404. JAK1 in HCC cells was clearly more elevated than in HHCs (Fig. 6d). To explain the relationship among circ9119, miR-26a, and JAK1 expression, cells underwent different transfection assays. First, cells were co-transfected with si-circ9119 and an miR-26a inhibitor (NC inhibitor). Thereafter, qRT-PCR and WB showed that the miR-26a inhibitor upregulated JAK1 in cells with circ9119 silencing (Fig. 6e–h). Meanwhile, transfection of the miR-26a/NC mimic was performed, and miR-26a upregulation led to reduced JAK1 expression, both at the mRNA and protein levels (Figs. 6i–l), indicating that miR-26a might be negatively correlated with JAK1 expression.

**Fig. 7:**
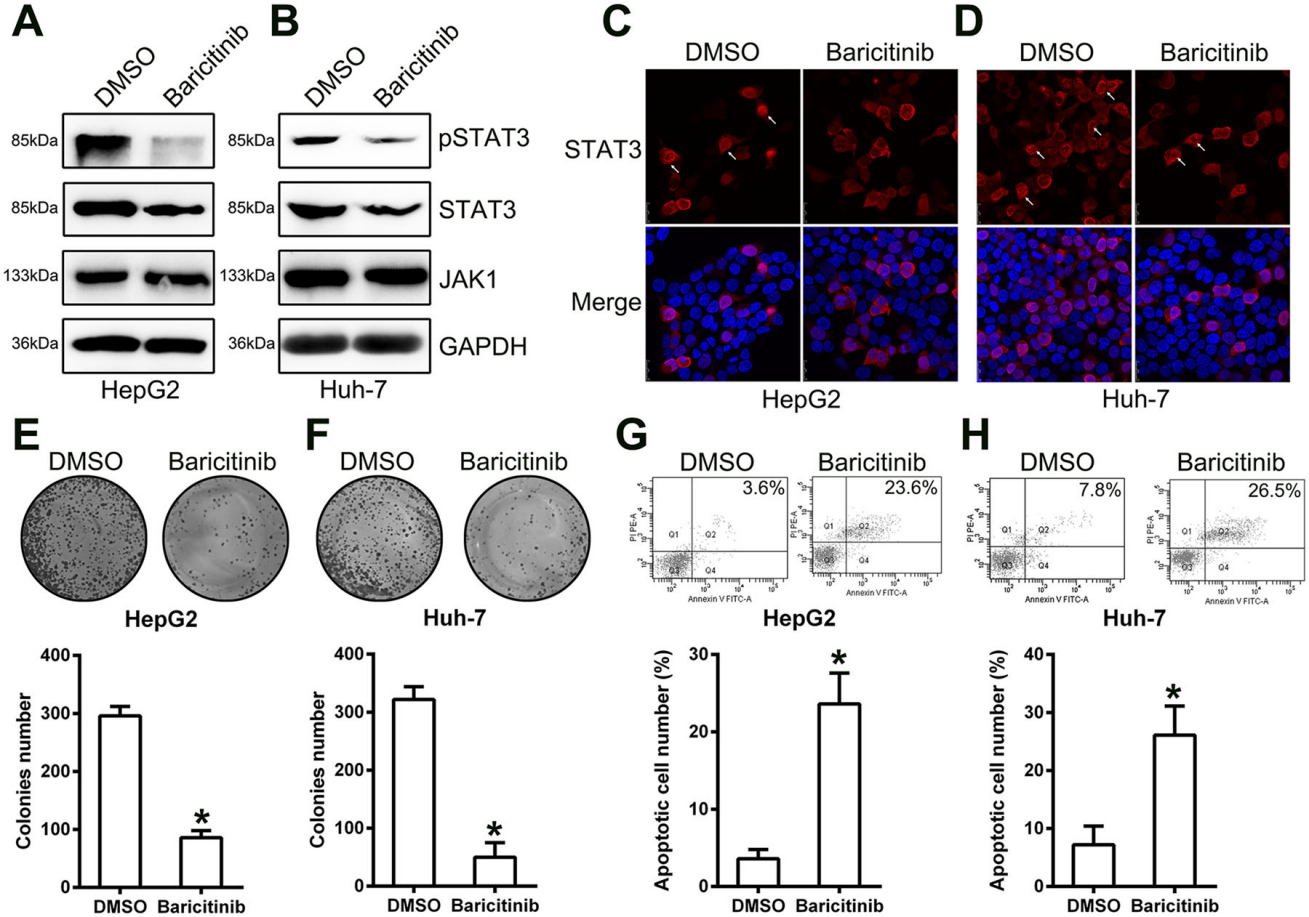
Administration of the JAK1 inhibitor, baricitinib, inhibits HepG2 and Huh-7 cell proliferation. TNM3 silencing reduces proliferation of SW579 cells. Cells were incubated with 2 μM baricitinib or 1% DMSO for 1 h. **a**, **b** Western blot analyses showing JAK1 and STAT3 expression, and STAT3 phosphorylation in HepG2 and Huh-7 cells, following incubation with baricitinib or DMSO. **c**, **d** Immunofluorescence assay showing the cellular localization of STAT3 in cells after incubation with baricitinib or DMSO. **e**, **f** Colony formation assay indicating HepG2 and Huh-7 growth rates, with baricitinib or DMSO treatment. **g**, **h** FCM evaluation of HepG2 and Huh-7 cell apoptosis with baricitinib or DMSO treatment. *p < 0.05 vs. indicated groups.

To elucidate the effect of JAK1 on cell properties, HepG2/Huh-7 cells were incubated with the JAK1 inhibitor, baricitinib, or 1% DMSO for 1 h, and then the colony formation assay and FCM were repeated. First, WB was carried out to confirm that HepG2/Huh-7 cells with baricitinib treatment showed downregulated downstream expression of JAK1 and STAT3 phosphorylation (Fig. 7a, b). Furthermore, we found that nuclear translocation of STAT3 was reduced upon incubation with baricitinib (Fig. 7c, d), indicating that the JAK1-STAT3 signal axis was blocked by baricitinib. We also observed, via colony formation assay, that baricitinib administration reduced HepG2 and Huh-7 cell growth (Fig. 7e, f). Additionally, there was an increase in apoptotic HepG2 and Huh-7 cells with baricitinib administration (Fig. 7g, h). These data showed that the JAK1-STAT3 pathway was an indispensable player in HCC cell survival.

### Circ9119 inhibition repressed tumorigenesis of HCC in vivo

**Fig. 8:**
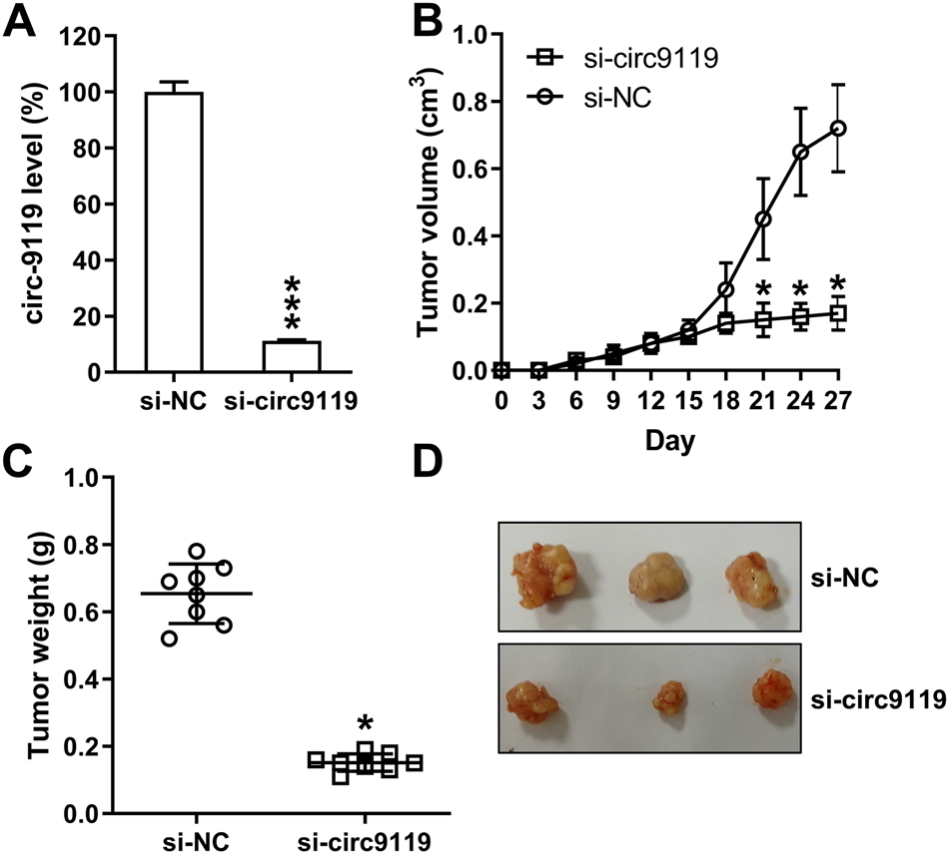
circ9119 silencing inhibits xenograft HCC tumor formation. **a** Expression of circ9119 in the tumors of each group using qRT-PCR analysis. Huh-7 cells infected with 1 × 10^7^ transduction unit (TU) lentiviral particles carrying si-NC or si-circ9119 were subcutaneously injected into mice (*N* = 8 per group). Mice were sacrificed and tumors were weighed at day 29 post inoculation. **b** Tumor growth curve during 28 days post inoculation. **c** Tumors weights after excision from each group. **d** Representative photographs of nude mice at day 28 post inoculation. **d** Data represent the mean ± SD. *p < 0.05, ***p < 0.001.

To determine the effect of circ9119 on xenograft pancreatic tumor formation, Huh-7 cells infected with lentiviral si-circ9119 or lentiviral si-NC were subcutaneously injected into BALB/c mice, and tumor growth was monitored daily. The expression of circ9119 was detected in the tumors of mice from each group. Circ9119 expression was confirmed to be silenced in the mice in the si-circ9119 group (Fig. 8a). These mice were sacrificed on day 28 after injection, and the pancreatic tissues were excised and weighed. Circ9119-silenced tumors grew at a much slower rate (Fig. 8b) and showed lower mean tumor weights than the tumors from the control group (Fig. 8c, d).

## Discussion

circRNAs are key regulators in multiple physiological and pathological processes and as miRNA sponges^25^. A crucial gene-regulating miRNA was recently identified on a couple of circRNAs^13^. circ9119 plays an essential role in receptive endometrium and testicular inflammation^3,24^. qRT-PCR results showed that both circ9119 levels and prostaglandin-endoperoxide synthase 2 (PTGS2) expression increase while miR-26a levels decrease in the receptive endometrium of dairy goats. circ9119 bound to and specifically decreased miR-26a levels, and miR-26a downregulated PTGS2 expression by binding to the target site to inhibit translation in dairy goat endometrial epithelial cells in vitro^3^. In another study, microarray data and quantitative real-time PCR showed that Toll-like receptor 3, circ9119, and retinoic acid inducible gene-I concentrations were elevated, whereas miR-136 and miR-26a were repressed. Inflammatory reactions were impaired as circ9119 was inhibited in separated Leydig and Sertoli cells that were treated with polyriboinosinic: polyribocytidylic acid (poly (I:C)). circ9119 bound to microRNAs, including miR-136 and miR-26a, thus repressing their expression. Inhibited miR-136 and miR-26a expression partially restored the expression of certain inhibited inflammatory cytokines when circ9119 was silenced^24^. Therefore, circ9119 was also a competing endogenous RNA, which bound to and isolated miR-26a, which coincided with our findings. Thus, circ9119 participated in HCC cell proliferation and apoptosis as a miR-26a sponge. Circ9119 silencing dampened HepG2 and Huh-7 cell viability and induced apoptosis in these two cell lines. Through bioinformatics analysis and luciferase activity reporter assays, we found that circ9119 could bind to miR-26a, and that the upregulation of circ9119 in HCC cell lines caused low miR-26a expression.

The established involvement of miRNAs in physiological processes varies from the timing of development, apoptosis, cell proliferation, and blood cell formation to nervous system patterning^26^. Increasing reports have documented that miR-26a inhibits colorectal^27^ and gastric^28^ cancer cells, while HCC cells multiply, migrate and invade^21–23^. Yang et al. found that downregulation of miR-26a is linked to higher HCC angiogenic potentiality. The demonstrated functions of miR-26a include greatly inhibiting the level at which HCC cells express vascular endothelial growth factor A and further suppressing, to some extent, HCC cell promotion of endothelial cell proliferation, metastasis, and capillary tube formation in vitro, which enhances HCC tumor angiogenesis in vivo^22^. Ma et al. showed that miR-26a regulates FBX011 to inhibit HCC cell multiplication, metastasis, and invasion^23^. Yang et al. determined that miR-26a functions in HCC cell growth and metabolic processes, and that HCC tissues frequently downregulate miR-26a, which might cause HCC recurrence and metastasis. MiR-26a obviously inhibits in vitro cell proliferation, metastasis, and invasion, as demonstrated by gain- and loss-of-function studies. Its induction of G1 arrest and promotion of HCC cell apoptosis were also shown. Other roles of miR-26a include specific suppression of human HCC tumor growth and spread in nude mice in vivo, markedly inhibiting STAT3 target genes (Bcl-2, Mcl-1, cyclin D1, and MMP2)^21^, which is consistent with the findings in this study. Although the function and mechanism of miR-26a as an essential regulator of tumor migration, invasion and viability of HCC cells has been shown, its upstream sensor is yet to be reported. This study showed that JAK1 is a novel protein tyrosine kinase targeted by miR-26a in HCC. qRT-PCR revealed that miR-26a expression was downregulated, and that miR-26a negatively regulated JAK1 in HCC cells. As confirmed by bioinformatics and luciferase reporter gene assays, JAK1 in HCC cells was directly targeted by miR-26a. Inhibition of miR-26a upregulated JAK1 expression and activated phosphorylation of STAT3, leading to its nuclear translocation. These data confirmed previous findings regarding miR-26a, suggesting a novel target and role of this miRNA in the JAK1-STAT3 pathway.

Some oncogenic signaling pathways are activated in varying human malignant conditions and the JAK-STAT pathway is among them^29^. Janus kinases (JAKs) are activated in this pathway when the corresponding ligands bind to cell-surface receptors. STATs are latent cytoplasmic transcription factors that are phosphorylated by JAKs and then relocate to the nucleus, where they bind to specific DNA elements, and guide gene transcription. The JAK-STAT pathway was initially believed to signal within cytokine or growth-factor receptors pathways^30^. STATs are a transcription factor family involved in cell proliferation, apoptosis, and other normal cellular events^31^. Growing evidence links STATs to apoptosis, of which STAT3 is a critical player^32^. As previously reported, human HCCs universally activate the JAK1/STAT3 pathway^33^ and it is crucial to activate the JAK1/STAT3 pathways for HCC progression. The continuous activation of these pathways in liver cancer may be due to suppression, and even apoptosis in certain HCC cell lines, by JAK/STAT pathway inhibitors. Our data showed that the JAK1/STAT3 pathway was upregulated and activated in HepG2/Huh-7 cells. circ9119 silencing caused deactivation of this pathway, along with increased apoptosis in HCC cells, while miR-26a inhibition led to the activation of the JAK1/STAT3 pathway, accompanied with reduced apoptosis. Administration of the JAK1/STAT3 pathway inhibitor, baricitinib, resulted in impaired HCC cell growth and induced apoptosis, suggesting potential applications of JAK/STAT inhibitors and reagents in human HCC therapy.

In summary, our study demonstrated that highly expressed circ9119 is a novel oncogene that promoted the viability of HCC cells through competitive interaction with the miR-26a-JAK1-STAT3 axis. This study provides a mechanistic understanding of the oncogenic role of circ9119 in HCC and indicates that circ9119, JAK1, or STAT3 might be important prognostic factors and biological targets for treating HCC.
